# Disrupting G6PD-mediated Redox homeostasis enhances chemosensitivity in colorectal cancer

**DOI:** 10.1038/onc.2017.227

**Published:** 2017-07-10

**Authors:** H-Q Ju, Y-X Lu, Q-N Wu, J Liu, Z-L Zeng, H-Y Mo, Y Chen, T Tian, Y Wang, T-B Kang, D Xie, M-S Zeng, P Huang, R-H Xu

**Affiliations:** 1 Sun Yat-sen University Cancer Center, State Key Laboratory of Oncology in South China, Collaborative Innovation Center for Cancer Medicine, Guangzhou 510060, China; 2 Department of Biomedical Engineering, School of Engineering, Sun Yat-sen University, Guangzhou 510060, China

## Abstract

Glucose-6-phosphate dehydrogenase (G6PD) is a key enzyme that generates NADPH to maintain reduced glutathione (GSH), which scavenges reactive oxygen species (ROS) to protect cancer cell from oxidative damage. In this study, we mainly investigate the potential roles of G6PD in colorectal cancer (CRC) development and chemoresistance. We discover that G6PD is overexpressed in CRC cells and patient specimens. High expression of G6PD predicts poor prognosis and correlated with poor outcome of oxaliplatin-based first-line chemotherapy in patients with CRC. Suppressing G6PD decreases NADPH production, lowers GSH levels, impairs the ability to scavenge ROS levels, and enhances oxaliplatin-induced apoptosis in CRC via ROS-mediated damage *in vitro. In vivo* experiments further shows that silencing G6PD with lentivirus or non-viral gene delivery vector enhances oxaliplatin anti-tumor effects in cell based xenografts and PDX models. In summary, our finding indicated that disrupting G6PD-mediated NADPH homeostasis enhances oxaliplatin-induced apoptosis in CRC through redox modulation. Thus, this study indicates that G6PD is a potential prognostic biomarker and a promising target for CRC therapy.

## Introduction

Altered redox status is a key biochemical feature that is frequently observed in tumor cells.^1^ In general, reactive oxygen species (ROS) at moderate levels is important for cell survival and tumorigenesis, but severe increases in ROS usually cause cell death.^1^ Cellular detoxification of harmful ROS commonly requires nicotinamide adenine dinucleotide phosphate (NADPH) as reducing equivalents.^2^ NADPH maintains the generation of reduced glutathione (GSH), which converts harmful H_2_O_2_ to water with glutathione peroxidase, resulting in glutathione disulfide.^3^ Perturbing NADPH production in cancer cells can have a positive therapeutic impact by increasing cell sensitivity to ROS and provoking apoptosis.^4^

Glucose-6-phosphate dehydrogenase (G6PD) catalyzes first step of pentose phosphate pathway (PPP) and plays key roles for NADPH generation.^5^ As the PPP is activated, G6PD is usually considered the pacesetter for NADPH production in cancer cells.^5^ Studies show that high expression of G6PD predicts poor clinical outcome in patients with various cancer, suggesting that G6PD may play critical roles in tumorigenesis.^6, 7^ More recently, several reports found that SIRT2-mediated deacetylation and SIRT5-mediated deglutarylation regulate G6PD activity to maintain cellular NADPH homeostasis.^8, 9^ However, the roles of G6PD in the development and chemoresistance of colorectal cancer (CRC) remain unclear.

CRC was considered the third most common diagnosed cancer in most Western countries.^10, 11^ Additionally, the incidence of CRC has rapidly increased in China over the past decade.^12^ Although numerous efforts have been made to improve treatment for CRC patients, most patients still die due to this disease as a result of therapeutic resistance to conventional agents and recurrence after resection. The chemotherapeutic agent oxaliplatin, a platinum-based antineoplastic agent, is generally considered as the first-line chemotherapy for CRC patients.^13^ Oxaliplatin induces DNA cross-links and excessive production of ROS, which cause cytotoxic effects by promoting apoptosis.^14, 15^ Given that oxaliplatin treatment induces ROS generation, we hypothesized that G6PD-mediated NADPH homeostasis may have the capacity to protect against oxidative stress, resulting in chemoresistance. Thus, we hypothesized that inhibition of G6PD-mediated antioxidant production would enhance oxaliplatin sensitivity in CRC through redox modulation.

In this study, we showed that high expression of G6PD predicts Furthermore, we show that RNAi-mediated G6PD knockdown enhances the sensitivity of CRC cells to oxaliplatin treatment and in cell based xenografts or PDX models. Thus, our study suggests that disturbing G6PD activity may be used as a therapeutic strategy for CRC in future.

## Results

### High expression of G6PD predicts poor prognosis in patients with CRC

**Figure 1:**
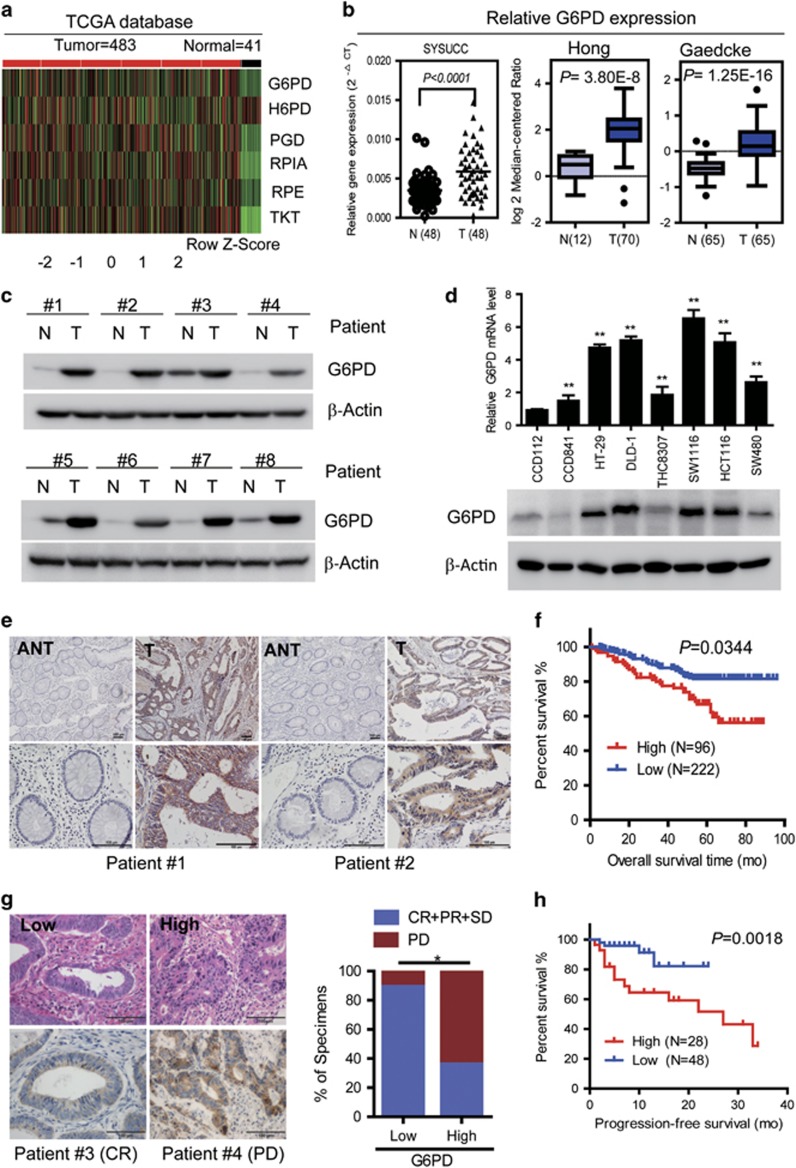
High expression of G6PD is associated with poor prognosis in colorectal cancer. (**a**) Expression profiling of PPP pathway related enzymes, including G6PD, H6PD, PGD, RPIA, RPE and TKT, in 483 primary CRC tissues and 41 adjacent normal tissues (TCGA). (**b**) G6PD is overexpressed in CRC tissues from our hospital (SYSUCC) analyzed by qPCR assay, and other microarray data sets available from Oncomine (https://www.oncomine.com//). (**c**) Immunoblotting analysis of G6PD expression in eight matched CRC tissues (T) and adjacent noncancerous tissues (N). (**d**) Immunoblotting and qPCR analysis of G6PD expression in two colorectal epithelial cells and six colorectal cancer cell lines. Data are presented as the mean±s.d. (*n*=3) **P<0.01 for indicated comparison (Student unpaired *t*-test). (**e**) Two representative cases show high expression of G6PD in human CRC tumor tissues (T) compared with adjacent normal tissues (ANT) tissues analyzed by IHC staining. (**g**) Two representative cases are shown (left) and percentage of specimens with low or high G6PD expression, relative to the response to FOLFOX or XELOX chemotherapy analyzed again by Pearson *Chi square* test (right). (**f**, **h**) The overall survival and progression-free survival curves of patients with low and high G6PD expression are generated using the Kaplan–Meier method and the log-rank test. PD, progressive disease; CR, complete response; PR, partial response; and SD, stable disease. Scale bar: 100 μm. β-Actin was used as a loading control.

PPP is best known for providing ribose phosphate and NADPH for biosynthesis redox homeostasis respectively.^5^ As shown in Figure 1a, the heatmap illustrates that most genes involved in PPP are overexpressed in CRC tumor tissues analyzed using The Cancer Genome Atlas (TCGA) database. To determine whether the rate-limiting enzyme G6PD is affected by colorectal tumorigenesis and chemoresistance, we first evaluated the expression of G6PD in CRC tissues. We found that G6PD mRNA was significantly increased in CRC tissues from our institute (SYSUCC) and Oncomine Microarray Database (Figure 1b, http://www.oncomine.org). Also, G6PD protein level was notably increased in eight representative tumor compared with adjacent normal tissues (Figure 1c). As expected, qPCR and immunoblotting analysis confirmed that G6PD mRNA and protein levels were increased in most detected CRC cells compared with the CCD112 and CCD841 cells (Figure 1d). Together, these results showed that G6PD expression is overexpressed in colorectal cancer.

To determine the clinical importance, we analyzed G6PD expression in 318 archived human CRC specimens. Representative IHC staining confirmed that G6PD expression was substantially increased in human paraffin-embedded CRC tissues (Figure 1e). Patients with low expression of G6PD had more favorable clinical outcomes, while patients with high expression of G6PD had shorter survival times (*P*=0.034; Figure 1f). Notably, a multivariate analysis showed that both G6PD expression and TNM stage were independent prognostic factors (Supplementary Table 1). To further determine the effects of G6PD expression on oxaliplatin efficacy, IHC staining for G6PD was performed on tumor tissues from 76 patients with advanced CRC treated with FOLFOX or XELOX regimens. Forty-three of 48 (89.5%) patients with low G6PD expression in their primary tumors benefited (CR+PR+SD) from chemotherapy, whereas only 10 of 28 (35.7%) patients with high G6PD expression benefited from the therapy (Figure 1g), suggesting that low G6PD expression predicts a favorable response to oxaliplatin-based chemotherapy. Strikingly, the progression-free survival (PFS) of patients with low G6PD protein expression was dramatically longer than that of patients with high G6PD expression (*P*=0.013; Figure 1h). On the basis of the above observations, we concluded that low G6PD protein expression predicts favorable clinical response to oxaliplatin-based chemotherapy and better survival.

### G6PD knockdown lowers NADPH levels and increases cellular susceptibility to oxidative stress

**Figure 2:**
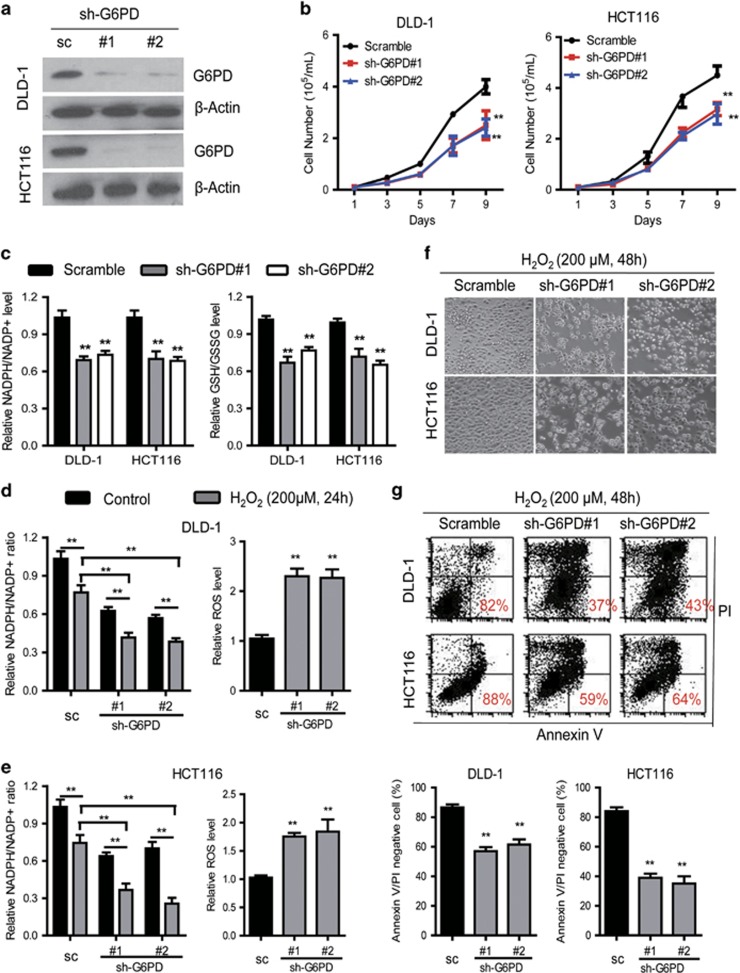
G6PD suppression disrupts NADPH homeostasis and increases CRC cell susceptibility to oxidative stress. (**a**) Immunoblotting analysis of G6PD in DLD-1 and HCT116 cells transduced with two specific shRNA. (**b**) Growth curves of DLD-1 and HCT116 cells with knockdown of G6PD. Scramble (sc): the lentiviral vector with a scrambled sequence that does not target any mRNA. (**c**) NADPH/NADP+ and GSH/GSSG levels were measured in DLD-1 and HCT116 cells with knockdown of G6PD. (**d**, **e**) NADPH/NADP+ and ROS levels were measured in indicated cells treated with H_2_O_2_ (200 μm) for 24 h. (**f**) Representative of morphology of indicated cells treated with H_2_O_2_ (200 μm) for 48 h. Original magnification, × 200. (**g**) Cell apoptosis was measured by Annexin-V/PI assay in G6PD-knockdown and control cells treated with H_2_O_2_ (200 μm) for 48 h (red numbers indicate subpopulation of cells negative for Annexin-V/PI). Data are presented as the mean±s.d. (*n*=3). **P<0.01 for indicated comparison (Student unpaired *t*-test).

Because G6PD acts as a guardian of antioxidative ability, we posited that intracellular NADPH reduction due to G6PD knockdown may impair resistance to oxidative stress and induce cellular apoptosis. To test this hypothesis, G6PD was stably knocked down in DLD-1 and HCT116 cells which exhibited high expression levels of G6PD using lentiviral G6PD shRNA (Figure 1d and 2a), and then evaluated the role of G6PD in regulating cellular NADPH homeostasis. Given the importance of G6PD in the production of nucleotide precursors, we first analyzed cell growth and proliferation of G6PD-knockdown CRC cells. As expected, knockdown of G6PD drastically reduced DLD-1 and HCT116 cell growth and proliferation (Figure 2b and Supplementary Figure S1A). Furthermore, G6PD-knockdown CRC cells reduced the NADPH/NADP+ or GSH/GSSG ratios and showed a substantial increase in ROS levels, confirming that G6PD is a key contributor to NADPH homeostasis in CRC cells (Figure 2c, Supplementary Figure S1B). Compared to the control cells, the G6PD-knockdown DLD-1 and HCT116 cells exhibited lower NADPH/NADP+ ratios following treatment with H_2_O_2_ (200 μM) for 24 h (Figures 2d and e). Taken together, G6PD knockdown lowers CRC NADPH/NADP+ ratio levels and increases cellular susceptibility to oxidative stress, which demonstrate that G6PD is required for redox homeostasis (Figures 2d and e).

When ROS accumulation overwhelms the cell detoxification capacity, ROS cytotoxicity damages the mitochondria and cell membranes, causing cell death.^1^ We further showed that cellular apoptosis and necrosis were also increased in G6PD-knockdown CRC cells treated with H_2_O_2_ (200 μM) for 48 h detected by the Annexin-V/PI assay (Figures 2f and g). Given that H_2_O_2_ induces DNA damage, the repair of which is dependent on the PPP to provide nucleotides. To test this notion, we exogenously added excess amounts of nucleotides, including dADP, dGDP, dCDP, and dUMP, to the culture medium of G6PD knockdown cells. This treatment partly reduced the percentage of apoptotic cells induced by H_2_O_2_ (Supplementary Figure S2A and B). Hydroxyurea (HU) inhibits ribonucleotide reductase which limits the availability of nucleotides for DNA synthesis and repair. The sensitivity of G6PD knockdown cell to HU was also investigated in the presence and absence of H_2_O_2_. Conversely, HU treatment significantly increased the sensitivity of G6PD knockdown cell to H_2_O_2_ (Supplementary Figure S2C). These findings demonstrate the key roles of G6PD in maintaining cellular NADPH homeostasis and promoting nucleotides synthesis for protecting cells from oxidative stress.

### G6PD knockdown enhances oxaliplatin-induced apoptosis in CRC cells

**Figure 3:**
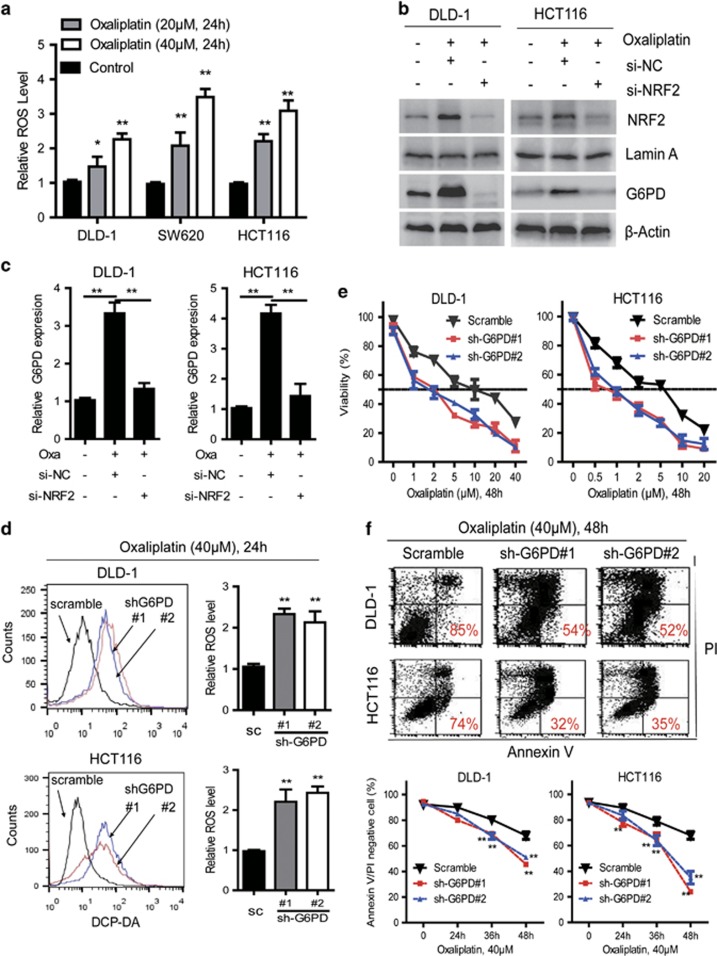
Knockdown of G6PD enhances oxaliplatin-induced apoptosis in CRC cells. (**a**) Increased ROS levels in CRC cells treated with oxaliplatin (20 μm or 40 μm) for 6 h. (**b**) Immunoblotting analysis of G6PD, Nrf2, Lamin A and β-Actin in DLD-1 and HCT116 cells treated with oxaliplatin or transfected with Nrf2 siRNA for 48 h. (**c**) qPCR analysis of G6PD expression in DLD-1 and HCT116 cells treated with oxaliplatin or transfected with Nrf2 siRNA for 48 h. (**d**) Increased ROS levels in G6PD-knockdown cells compared with control cells treated with oxaliplatin (40 μm) for 24 h, and representative histograms are shown. (**e**) The cell viability were measured using MTS assays in G6PD-knockdown and control cells treated with oxaliplatin for 48 h. (**f**) Cell apoptosis was measured by Annexin-V/PI assay in G6PD-knockdown and control cells treated with oxaliplatin (40 μm) for the indicated period of time. Numbers indicate percentages of live cells. Data are presented as the mean±s.d. (*n*=3). **P<0.01 for indicated comparison (Student unpaired *t*-test).

Cancer chemotherapy drugs are known to induce cell apoptosis and ROS formation. Accordingly, the cell generally induces a compensatory increase in cellular antioxidant activity to avoid potential ROS injury.^1, 16^ We found that the oxaliplatin treatment also induced significant increase of cellular ROS levels in CRC cells (Figure 3a). Responding to ROS, NRF2 plays a vital role in transcriptionally regulating the expression of antioxidants genes including G6PD.^17, 18^ Immunoblotting analysis indicated that oxaliplatin treatment induced NFR2 activation and G6PD expression in CRC cells (Figure 3b). Also, RNAi depletion of NRF2 decreased G6PD mRNA and protein levels in these oxaliplatin-treated CRC cells (Figures 3b and c). Therefore, we posited that G6PD knockdown may enhance oxaliplatin sensitivity in CRC through redox modulation. Then, we tested the effects of G6PD knockdown on the efficacy of oxaliplatin in CRC cells. As shown in Figure 3d, G6PD knockdown specifically increased ROS levels in CRC cells treated with 40 μM oxaliplatin for 24 h. Accordingly, G6PD knockdown increased oxaliplatin sensitivity in CRC cells as determined by MTS assays (Figure 3e). The IC_50_ values for the cells transfected with shRNA#1 and siRNA#2 were decreased compared with that of the control as shown in Figure 3e. Annexin-V/PI assays also showed that treatment with 40 μM oxaliplatin resulted in more apoptotic and necrotic in G6PD-knockdown DLD-1 and HCT116 cells (Figure 3f). We exogenously added excess amounts of nucleotides to the culture medium of G6PD knockdown cells treated with oxaliplatin. This treatment partly reduced the percentage of apoptotic cells induced by oxaliplatin (Supplementary Figure S2D and E). Conversely, HU treatment significantly increased the sensitivity of G6PD knockdown cell to oxaliplatin (Supplementary Figure S2F). Most importantly, oxaliplatin-induced apoptotic cells were significantly reduced upon addition of the antioxidant NAC (N-acetyl cysteine, a GSH precursor) in G6PD-knockdown CRC cells (Supplementary Figure S3A). This indicates that accumulation of H_2_O_2_ contributes to the cytotoxic effect induced by oxaliplatin treatment plus G6PD inhibition. Taken together, these results suggested that G6PD plays critical roles in determining the sensitivity of CRC cells to oxaliplatin.

### G6PD knockdown enhances oxaliplatin-induced cell death through ROS-mediated damage

**Figure 4:**
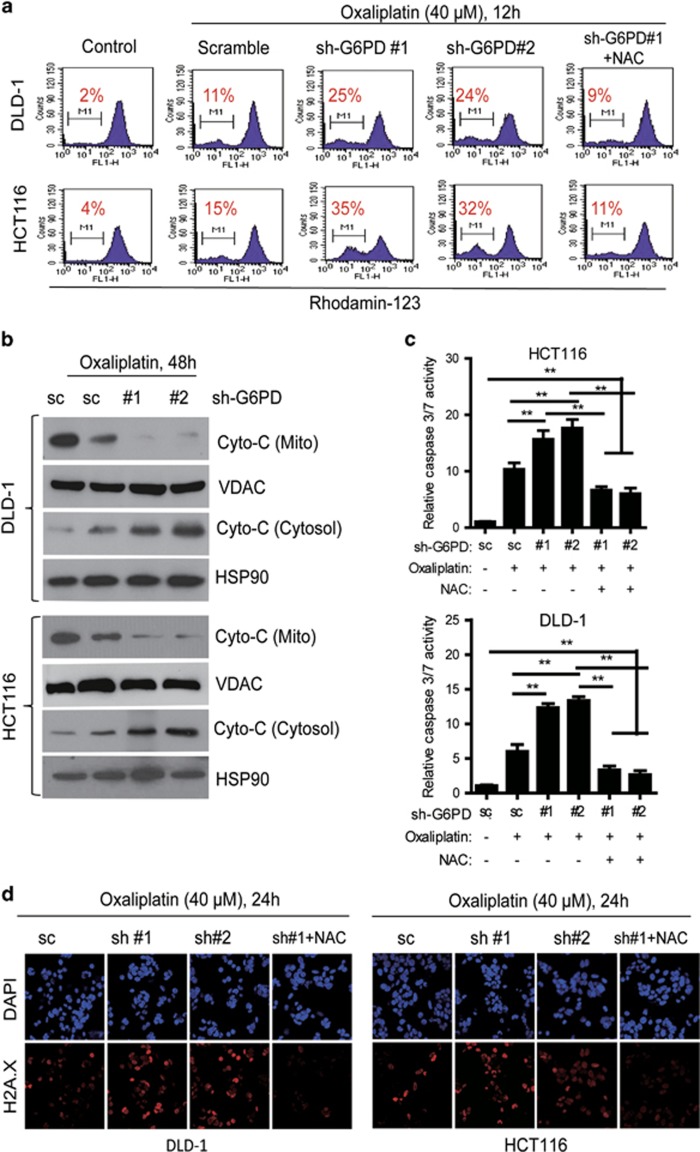
G6PD suppression enhances oxaliplatin-induced apoptosis through ROS-mediated damage. (**a**) Comparison of the mitochondrial transmembrane potential in the indicated cells after treatment of 40 μM oxaliplatin for 12 h, as measured by the potential-sensitive probe rhodamine-123. (**b**) Immunoblotting analysis of Cyto-C in subcellular fractions isolated from the indicated cells after treatment of oxaliplatin (40 μM) for 48 h. HSP90 and VDAC were used as markers of cytoplasm and mitochondria. (**c**) Caspase 3/7 activity was measured in indicated cells treated with oxaliplatin (40 μm) or a combination with the antioxidant NAC (5 mM) for 48 h. The results are presented as a fold increase relative to the untreated sample. Data are presented as the mean±s.d. (*n*=3). (**d**) Immunofluorescence staining of γH2A.X formation (red fluorescence) in indicated cells treated with oxaliplatin (40 μM) or a combination with the antioxidant NAC (5 mM) for 24 h. Nuclei were stained with DAPI (blue fluorescence). **P<0.01 for indicated comparison (Student unpaired *t*-test).

Excessive ROS production is known to damage the mitochondrial membrane, enhance the release of cytochrome c and trigger cell apoptosis.^19^ To clarify the underlying mechanisms, we further determined whether G6PD knockdown could increase cytochrome c release and activate the caspases in oxaliplatin-treated CRC cells. Treated cells were labeled with rhodamine-123, and tested the mitochondrial membrane potential (MMP). As shown in Figure 4a, 40 μM of oxaliplatin had a minimal effect on the MMP after 12 h of incubation, but G6PD knockdown increased the loss of the potential in the oxaliplatin-treated CRC cells. Most importantly, the effect of the G6PD knockdown and oxaliplatin combination was reversed by pretreatment with the antioxidant NAC (5 mM) (Figure 4a). Immunoblotting analysis of the mitochondrial and cytosolic protein fractions showed that G6PD knockdown increased cytochrome c release in the oxaliplatin-treated CRC cells (Figure 4b). Measurement of caspase 3/7 activity further confirmed that G6PD knockdown increased oxaliplatin sensitivity in CRC cells after a 48 h incubation and that supplementation with NAC prevented cell death (Figure 4c). The γH2A.X foci formation is a hallmark induced by platinum drugs treatment, including cisplatin and oxaliplatin.^20^ Both DLD-1 and HCT116 cells were evaluated for γH2A.X foci formation (Figure 4d). Cells treated with 40 μM oxaplatin alone for 24 h exhibited litter γH2A.X signal, however silencing G6PD resulted in more intensity of γH2A.X detected by immunofluorescence analysis (Figure 4d). Immunoblotting analysis of γH2A.X expression also showed similar results in these tested cells (Supplementary Figure S3B). These results indicate that accumulation of H_2_O_2_ contributes to the cytotoxic effect induced by G6PD knockdown plus oxaliplatin treatment, and depletion of G6PD enhances oxaliplatin-induced cell death through ROS-mediated damage.

### G6PD knockdown enhances oxaliplatin efficacy in cell based xenografts.

**Figure 5:**
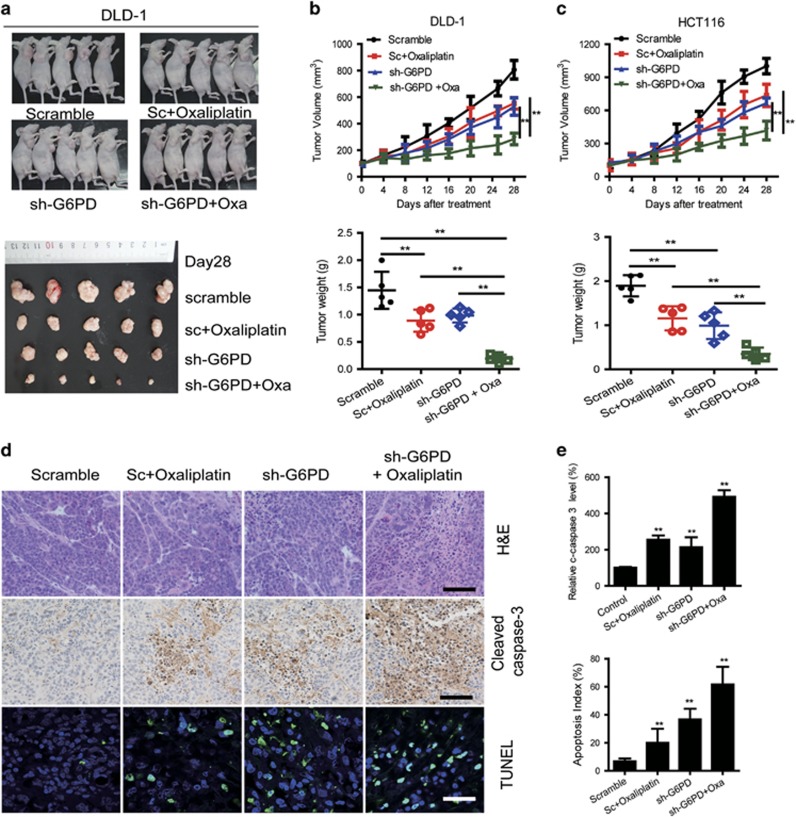
Knockdown of G6PD enhances oxaliplatin efficacy *in vivo.* (**a**) G6PD knockdown or control DLD-1 cells (2 × 10^6^) were subcutaneously injected into the nude mice. The mice were treated with oxaliplatin when the tumor volume reached 100 mm^3^ in the control group, and images of tumors derived from indicated group are shown. (**b**, **c**) The tumor growth curves and tumor weights of indicated group mice were measured and recorded. (**d**) Paraffin-embedded tumor sections derived from DLD-1 cells bearing mice were stained with H&E or cleaved caspase 3 antibodies (scale bar: 100 μm); apoptotic cells were visualized and quantified by TUNEL staining (green) and counterstained with DAPI (blue) (scale bar: 10 μm). (**e**) Quantification of cleaved caspase 3 levels and apoptotic index (TUNEL staining) in CRC tumors was shown. Data are presented as the mean±s.d. (*n*=5). **P<0.01 for indicated comparison (Student unpaired *t*-test).

To further investigate whether G6PD knockdown affects oxaliplatin sensitivity *in vivo*, equal amounts of log-phase G6PD knockdown or control HCT116 or DLD-1 cells (2 × 10^6^) were injected into mouse flank subcutaneously. Ten days later, oxaliplatin (5 mg/kg) was administered every 4 days for 4 weeks. The G6PD-knockdown group treated with oxaliplatin proved a large reduction in tumor weight and tumor growth (Figure 5a). The tumors derived from the HCT116 or DLD-1 cells with G6PD knockdown were significantly more sensitive to oxaliplatin than the HCT116 or DLD-1 vector control-derived tumors. The oxaliplatin-mediated growth inhibition rates of the G6PD knockdown tumors and the control tumors were 86.7 and 45.3% in HCT116 cells and 81.4 and 39.0% in DLD-1 cells, respectively (Figures 5b and c). Furthermore, the combination of oxaliplatin treatment and G6PD knockdown resulted in more apoptosis by comparison with oxaliplatin or G6PD knockdown, as determined by IHC (cleaved caspase 3-positive) and TUNEL staining (Figure 5d). Overall, the combination of oxaliplatin treatment with G6PD knockdown exhibited obviously synergistic effects for CRC treatment in cell line-based xenografts.

### Delivery of G6PD shRNA with poly (*amino-co-ester*) polyplexes enhances oxaliplatin efficacy in PDX models

**Figure 6:**
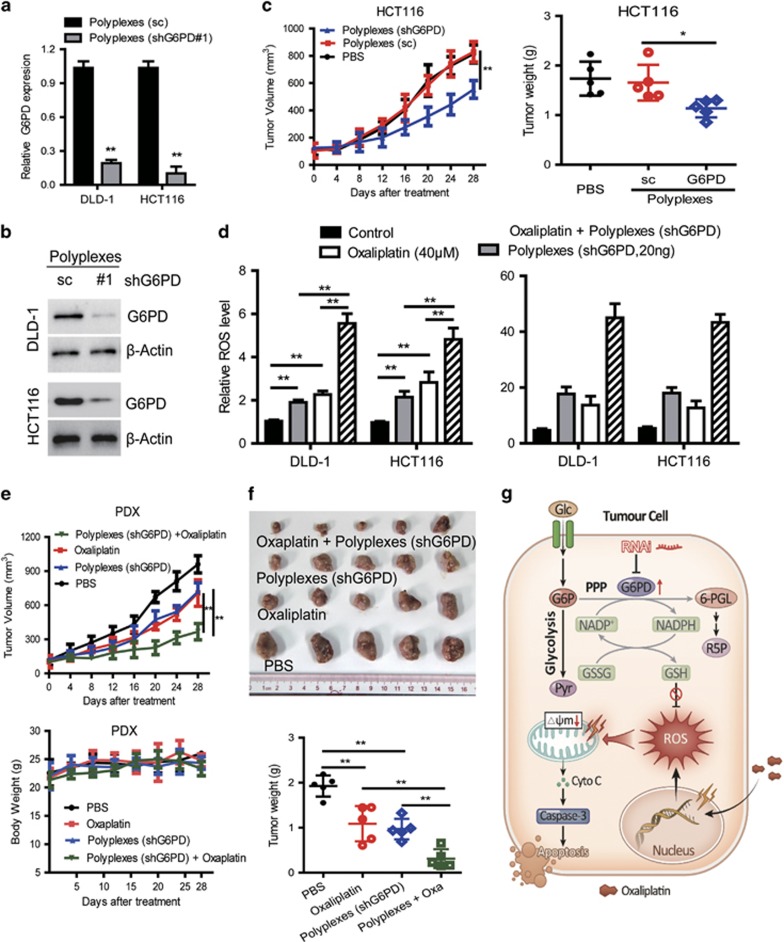
Delivery of G6PD shRNA with PPMS polyplexes enhances oxaliplatin efficacy in CRC cells and patient-derived xenograft (PDX) model. (**a**) qPCR analysis of G6PD expression in DLD-1 and HCT116 cells transfected with PPMS polyplexes with G6PD shRNA or scramble plasmid DNA. (**b**) Immunoblotting analysis of G6PD expression in indicated cells transfected with PPMS polyplexes with G6PD shRNA or scramble plasmid DNA. (**c**) The tumor growth curves and the tumor weights of the mice injected with HCT116 cells were measured and recorded for each group throughout the experiment (*n*=5). Polyplexes were administrated through tail vein injection every 4 days, at the dose of 1.7 mg per mouse, for 4 weeks. The dose was chosen based on the maximum amount of the polymer that can be used in 200 μl buffer solution for injection. (**d**) The cellular ROS levels and the percentage of apoptotic cells were measured in indicated cells treated with oxaliplatin, transfected with polyplexes (shG6PD) or their combination. (**e**) The tumor growth curves and the weights of the PDX mice were measured and recorded for each group throughout the experiment (*n*=5). (**f**) PDX mice were weighed every 4 days for 28 days to estimate toxicity. (**g**) Proposed working model of this study. Data in (**a**, **d**) are presented as the mean±s.d. (*n*=3). **P<0.01 for indicated comparison (Student unpaired *t*-test).

PDX tumors have been suggested as a more realistic experimental and preclinical model.^21^ In addition to cell line-based xenograft models, PDX models were also performed to assess clinical benefits of targeting G6PD. Non-viral vectors for gene delivery have become a rapid-developing field recently.^22^ We have established a new gene delivery system based on cationic poly(ω-pentadecalactone-*co*-*N-*methyldiethyleneamine-*co*-sebacate) (PPMS) polyplex with high gene transfection capability.^23, 24^ The modified G6PD shRNA#1/PPMS polyplexes were prepared according to our previous study.^31^ First, the knockdown efficiency was evaluated in CRC cells using PPMS polyplexes loaded with G6PD shRNA#1. The results indicated that the PPMS/shG6PD polyplexes resulted in a significant downregulation of G6PD on both mRNA level and protein level (Figures 6a and b). Also, the tail vein injection of PPMS polyplexes silenced G6PD expression and yielded effective inhibition of tumor growth compared to the scramble and PBS control groups *in vivo* (Figure 6c). To test our hypothesis, we tested the effects of G6PD knockdown by PPMS polyplexes on the efficacy of oxaliplatin in CRC cell lines and PDX models with high expressed G6PD. We found that the combination of polyplexes (shG6PD) increased oxaliplatin-induced ROS levels and cell death in tested HCT116 and DLD-1 cells (Figure 6d). Furthermore, the combination of oxaliplatin treatment and G6PD knockdown by PPMS polyplexes resulted in the more potent anti-tumor activity compared with oxaliplatin or G6PD knockdown alone, which was evident by the significant reduced PDX tumor growth and tumor weight (Figures 6e and f). This result indicates a similar tendency to the cell line-based xenograft models, and highly suggests the potential of G6PD as a therapeutic target for CRC treatment and overcoming oxaliplatin chemoresistance. As shown in Figure 6g, this study suggests that disrupting G6PD-mediated NADPH homeostasis enhances oxaliplatin-induced apoptosis in CRC through redox modulation.

## Discussion

G6PD is considered to play pro-oncogenic roles based on its overexpression in various tumors.^7, 25^ For all we know, this study is the first to demonstrate that G6PD-mediated cellular NADPH homeostasis is involved in the oxaliplatin treatment effects for CRC. ROS have been shown to mediate chemosensitivity in cancer.^16, 26^ Oxaliplatin, a third-generation platinum analog, functions by preventing DNA replication.^27^ Our results indicate that the cellular ROS is important for the oxaliplatin-induced cytotoxicity. The increased ROS generation usually induces a compensatory increase in NRF2-mediated cellular antioxidant activity to protect cells from ROS injury, resulting in chemoresistance. Finally, whether the cells die or survive is decided by the prooxidant and antioxidant capacities of the cancer cells. Excessive levels of ROS stress can exhaust the antioxidant capacity and promote apoptosis by increasing the ROS stress level beyond a threshold.^28^ Previous studies demonstrated that logical combinations of ROS-modulating compounds with chemotherapeutic drugs may further enhance therapeutic activity.^29, 30^ Recently, Yun *et al.*^31^ found that vitamin C–induced ROS inhibits GAPDH activity by NAD+ depletion, ultimately leading to cell death in BRAF and KRAS mutant CRC cells. Conversely, another study showed that inhibition of ROS generation by cetuximab impairs oxaliplatin-induced cell death in preclinical models.^15^

The current trend in chemotherapy is personalized medicine, and identifying novel biomarkers and potential therapeutic targets is a major challenge. Here, we systematically explored the effect of targeting G6PD on oxaliplatin efficacy in CRC in cells, cell line-based xenografts or PDX models with high expression of G6PD, suggesting this treatment approach could be used for CRC. To evaluate the therapeutic effect on G6PD in PDX models, we also used a nano-sized cationic polymeric gene delivery system for G6PD shRNA delivery based on our previous reports.^23, 24^ Our finding clearly demonstrated that the biodegradable PPMS polyplexes could effectively deliver G6PD shRNA to PDX models through tail vein injection and the downregulation the expression of G6PD could significantly enhances the therapeutic efficacy of oxaliplatin. This study also indicates that the non-vial nano gene delivery vector could be applied to CRC treatments in future. Beside, recent study showed that aspirin inhibits G6PD activity in CRC cells through acetylation.^32^ Additionally, a novel clinical-stage radiosensitizer and chemosensitizer RRx-001 exerts the anti-proliferative effects through inhibiting G6PD activity in human tumor cells.^33^ Future study should focus on the development of selective inhibitors for G6PD and the combinatorial effects with clinical chemotherapy drugs.

In conclusion, understanding of the precise roles of G6PD in advanced CRC may allow it as a prognostic biomarker for patient response to oxaliplatin-based chemotherapy and aid in the development of novel therapeutic strategies.

## Materials and Methods

### Cell culture

The immortalized colon epithelial cells (CCD112, CCD841) and CRC cells (SW1116, HT-29, THC8307, DLD-1, HCT116 and SW480) were obtained from the ATTC (Manassas, VA, USA) and cultured as recommended. All CRC cells were negatively detected for mycoplasma contamination before use. All cell lines were authenticated based on STR fingerprinting at the Department of Medicine Lab of Forensic Medicine in our institution (Guangzhou, China).

### Reagents and antibodies

The following reagents were purchased: oxaliplatin, hydroxyurea (HU) (Selleck, Houston, USA); Deoxynucleoside triphosphate set (dNTP, including dATP, dCTP, dGTP and dTTP) (Sigma-Aldrich, MO, USA); DCFH-DA, Rhodamine-123 and DAPI (Invitrogen, Carlsbad, CA, USA); GSH/GSSG-Glo assay kit, NADPH/NADP-Glo assay kit and Caspase 3/7 Glo assay kit (Promega, Wisconsin, USA). The following antibodies were also purchased: G6PD (#ab133525), Lamin A (#ab8980), γH2A.X (#ab2893) (Abcam, Cambridge, USA); Cleaved caspase 3 (#9664), Cytochrome c (#4280), VDAC (#4866), NRF2 (#12721), HSP90 (#4877) and β-Actin (#3700) (Cell Signaling Technology, Beverly, USA).

### Flow cytometry analysis

The intracellular ROS levels, cell apoptosis and the mitochondrial transmembrane potential (MMP) were determined by flow cytometry (FACSCalibur, Becton Dickinson) as described in previous publication.^16^ Briefly, DLD-1 or HCT116 cells were incubated with 3 μM DCFH-DA or 1 μM rhodamine-123 for 60 min for ROS and MMP detection. For cell apoptosis analysis, CRC cells were incubated with H_2_O_2_ (200 μM) or oxaliplatin (40 μM) for 48 h, harvested and resuspended in 500 μl of staining buffer plus propidium iodide (PI) and Annexin-V-FITC. The percentage of cells undergoing apoptosis were evaluated by flow cytometry.

### Cell transfection and lentivirus production

The small interfering RNA (siRNA) targeting NRF2 (targeting sequence: uaaaguggcugcucagaau) were synthesized by RiboBio (Guangzou, China). Transfection was conducted using DharmaFECT reagent (GE Dharmacon, Lafayette, USA) as recommended. The lentiviruses containing G6PD shRNA were purchased from GenePharma (Shanghai, China). The infection was conducted and stable cell lines (HCT116 or DLD-1 cells) expressing G6PD shRNAs were selected for 2 weeks with 3 μg/ml puromycin. The sequences targeting G6PD were gccttccatcagtcggata (#1) and cctcatggtgctgagattt (#2).

### Immunohistochemistry analysis

Immunohistochemistry (IHC) analysis was conducted with standard procedures as described previously.^34^ The prepared slides were incubated with antibodies against G6PD (1:200 dilution) or cleaved caspase 3 antibody (1:2000 dilution). Two gastrointestinal pathologists assessed the IHC staining based on the proportion (0, <25% 1, 25–50% 2, 50–75% 3, 75–100%) of the G6PD stained cells and the extent of the G6PD staining from no staining, weak, moderate to strong (0, 1, 2, 3). The expression levels of G6PD was considered high (⩾ 4) or low (<4) based on the final scores generated by multiplying the staining proportion scores with staining extent scores.

### *In vivo* therapeutic study

CRC cell line-based xenograft and patient-derived xenograft (PDX) models were performed to assess the clinical benefits of targeting G6PD. The BALB/c nude mice (8 week old, female) were obtained from the Guangdong Province Laboratory Animal Center. PDX models were obtained as previously published reports.^35, 36^ Randomization was conducted. The cell line-based experiments were designed as follows: the mice were randomly divided into the Scramble, sc+oxaliplatin, shG6PD, and shG6PD+oxaliplatin groups (five mice per group), and equal amounts of G6PD-knockdown or control HCT116 or DLD-1 cells (2 × 10^6^ cells per mice) were injected into the mouse flank subcutaneously. Oxaliplatin was administered by intraperitoneal (i.p.) injection (5 mg/kg) every 4 days for four weeks. Tumor sizes were measured every 4 days using an unblinded manner as described previously.^37^ PDX tumors with high expressed G6PD were generated and implanted. When the tumor had grown to an appropriate volume, the tumor-bearing mice were assigned to four groups (five mice per group) randomly;^32^ control group: received 200 μl PBS every 4 days;^13^ oxaplatin group: received oxaplatin at 5 mg/kg every 4 days by i.p. injection;^17^ The polyplexes (shG6PD) group: delivery of G6PD shRNA#1 with poly (amino-*co*-ester) polyplexes every 4 days by intravenous (i.v.) injection;^26^ combination group. The animals were treated for 4 weeks. All animal studies were approved by the Institutional Animal Care and Use Committee at our institution.

### Patient information

The archived CRC tissue specimens (*N*=318) were obtained from fresh surgical specimens between January 2010 and July 2013 at our hospital (Guangzhou, China). Seventy six patients with histopathologically and clinically diagnosed metastatic CRC were enrolled in our study from 2000 to 2007. All patients received XELOX (capecitabine and oxaliplatin) or FOLFOX (oxaliplatin, leucovorin and fluorouracil) regimens at our hospital. The use of these clinical CRC specimens was approved by our Institutional Research Ethics Committee. The prior patient consent was also obtained from each patient. Detailed information is described in our previous publication.^38^

The details for RNA extraction and qPCR analysis, subcellular fractionation, immunoblotting analysis, Immunofluorescence analysis and colony formation assay are described in the Supplementary Materials.

### Statistical analysis

All experiments were repeated three times or more and data are presented as mean±s.d. evaluated using Student's *t*-test (unpaired, two-tailed). The variance between the groups that are being statistically compared is similar. Sample size was chosen based on the need for statistical power. Survival curves were generated using the Kaplan–Meier method and compared using the log-rank testing. The independent prognostic factors were identified by the Cox proportional hazard regression model. Differences reached statistical significance with *P*<0.01 (**) and *P* <0.05 (*) analyzed by GraphPad Prism 5 (La Jolla, CA, USA).
