# Supplementary material for: Disrupting G6PD-mediated Redox homeostasis enhances chemosensitivity in colorectal cancer
Source: Oncogene. 2017 Jul 10;36(45):6282–92. doi: 10.1038/onc.2017.227 (PMC5684443; doi:10.1038/onc.2017.227)
Supplement: Supplementary Information [file onc2017227x1.docx]

**SUPPLEMENTARY INFORMATION**

**SUPPLEMENTARY TABLE 1.**

**Univariate and multivariate analyses of various potential prognostic factors in CRC patients**

|  | | Univariate analysis | | | | Multivariate analysis | | | |
| --- | --- | --- | --- | --- | --- | --- | --- | --- | --- |
|  |  | HR (95% CI) | | *P* | HR (95% CI) | | | *P* |  |
| Age (<60/≥60) | 1.02-2.82 | | 0.042^*^ | | 1.09-3.03 | | 0.021^*^ | |  |
| Gender (male/female) | 0.99-1.04 | | 0.061 | | - | | - | |  |
| Differentiation  (well, moderate/poor) | 0.00-56.6 | | 0.400 | |  | |  | |  |
| Tumor depth (m, sm, mp/ss, se, si) | 0.65-4.05 | | 0.298 | |  | |  | |  |
| Tumor size (≥5cm/<5cm) | 0.48-1.32 | | 0.378 | | - | | - | |  |
| Lymph node invasion (present/absent)  CEA(abnormal/normal)  CA199(abnormal/normal) | 3.04-10.7  0.97-2.60  1.49-4.17 | | 0.000  0.067  0.001 | | 1.30-3.67 | | 0.003^*^ | |  |
| TNM Stage(I-II/III-IV) | 3.48-14.3 | | 0.000 | | 3.45-14.3 | | 0.000^*^ | |  |
| G6PD protein (high/low) | 1.03-2.84 | | 0.037 | | 1.10-3.03 | | 0.034^*^ | |  |

HR: hazard ratio; CI: confidence interval; m: tumor invasion of mucosa; sm: submocosa; mp: muscularis propria; ss: subserosa; se: serosa penetration; si: invasion to adjacent structures. **P* < 0.05.

**SUPPLEMENTARY MATERIAL AND METHODS**

**RNA extraction and qPCR assay**

Total RNA was extracted from CRC tissues or cells using Trizol (Invitrogen, Carlsbad, USA). The complementary DNA (cDNA) was reverse-transcribed using the PrimeScript RT Mix (Bio-Rad, Hercules, CA). Then, the real-time PCR was performed with SYBR reagents (Bio-Rad) to measure the levels of G6PD. Expression of β-Actin was also measured and used as the internal control for normalization. The forward and reverse primer sequences are as follows: G6PD, forward: 5ˈ-cgaggccgtcaccaagaac-3ˈ; reverse: 5ˈ-gtagtggtcgatgcggtaga-3ˈ; β-Actin, forward: 5ˈ-catgtacgttgctatccaggc-3ˈ; reverse: 5ˈ-ctccttaatgtcacgcacgat-3ˈ.

**Subcellular fractionation and immunoblotting analysis**

CRC cells were harvested using trypsin-EDTA and washed twice with cold PBS. Cytosolic, mitochondrial and nuclear fractions of CRC cells were prepared using cell fractionation kit (#ab109719) as described in the protocol (Abcam, Cambrige, UK). Fractions were analyzed immunoblotting assay as described previously ([Ju et al 2017](#_ENREF_2)). HSP90, Lamin A and VDAC were used as markers of cytoplasm, nucleus and mitochondria in immunoblotting analysis.

**Immunofluorescence and confocal microscopy**

Logarithmic growth CRC cells (DLD-1 or HCT116 cells) were seeded in Glass Bottom Cell Culture Dish (NEST, Wuxi, China) overnight and treated with oxaliplatin for indicated time. Then, cells were washed with PBS for 5 min twice and fixed with 3.7% paraformaldehyde and permeabilized with 0.25% Triton X-100. The samples were then incubated with 5% BSA for 30 min at room temperature (RT) followed by incubation with γH2A.X antibody (Abcam, Cambrige, UK) at 4 °C overnight. Samples were then incubated with AlexaFluor-555 goat anti-rabbit antibody (Invitrogen, Carlsbad, CA, USA) for at RT 1 h. samples were stained with 400 ng/ml DAPI in PBS for 5 min at RT. Images were taken by NIKON Eclipse TE2000 confocal microscope and analyzed by using Nikon EZ-C1 software.

**Cell viability and colony formation assay**

CRC cells (3×10^3^ cells/well) were seeded in a 96-well plates overnight. After 72 h of exposure to oxaliplatin (1μM, 2μM, 5μM, 10μM, 20μM, 40μM), cell viability was determined using the MTS assay. For the colony formation assay, CRC cells (DLD-1 or HCT116) were seeded into 6-well plates at a density of 500 cells/well. The cells were cultured for 14 days before they were fixed in formalin and stained with crystal violet as described previously ([Ju et al 2016](#_ENREF_1)). The colonies were counted and relative colony number was shown.

**REFERENCES**

Ju HQ, Lu YX, Chen DL, Tian T, Mo HY, Wei XL *et al* (2016). Redox Regulation of Stem-like Cells Though the CD44v-xCT Axis in Colorectal Cancer: Mechanisms and Therapeutic Implications. *Theranostics* **6:** 1160-1175.

Ju HQ, Ying H, Tian T, Ling J, Fu J, Lu Y *et al* (2017). Mutant Kras- and p16-regulated NOX4 activation overcomes metabolic checkpoints in development of pancreatic ductal adenocarcinoma. *Nat Commun* **8:** 14437.

**SUPPLEMENTARY FIGURE LEGENDS**

**Supplementary Figure 1.** **RNAi-mediated G6PD knockdown decreases CRC cell growth and proliferation *in vitro*.** (A) Images and quantification of colony formation by indicated CRC cells with knockdown of G6PD. (B) Representative histograms shown the increased ROS levels in G6PD-knockdown CRC cells. Data are presented as the mean ± SD (n=3). ***P* < 0.01 for indicated comparison (Student unpaired *t*-test).

**Supplementary Figure 2. The effects of exogenous supply of nucleotides on G6PD depletion-induced apoptosis under oxidative stress.** (A-B) The effects of exogenous nucleotide supply, including dADP, dGDP, dCDP, and dUMP (300 µM), on the percentage of apoptotic cells induced by H_2_O_2_ (200 µM) for 48h. Cell apoptosis was measured by Annexin-V/PI assay and numbers indicate percentages of live cells. dNTPs were added to the culture media 24h before. (C) The sensitivity of G6PD knockdown cell to HU (2mM) was also investigated in the presence and absence of H_2_O_2_ (200 µM, 48h) by Annexin-V/PI assay. (D-E) The effects of exogenous nucleotide supply, including dADP, dGDP, dCDP, and dUMP (300 µM), on the percentage of apoptotic cells induced by oxaliplatin (40 µM) for 48h as described above. (F) The sensitivity of G6PD knockdown cell to HU (2mM) was also investigated in the presence and absence of oxaliplatin (40 µM, 48h) by Annexin-V/PI assay. dNTP, deoxynucleoside thiphosphate. Data are presented as the mean ± SD (n=3). ***P* < 0.01 for indicated comparison (Student unpaired *t*-test).

**Supplementary Figure 3.** **G6PD suppression enhances oxaliplatin-induced apoptosis through ROS-mediated damage**. (A) Pretreatment with 5 mM NAC for 2 h significantly inhibited the cell death in the indicated CRC cells induced by oxaliplatin (40µM, 36 h) treatment. Data are presented as the mean ± SD (n=3). ***P* < 0.01 for indicated comparison (Student unpaired *t*-test). (B) Immunoblotting analysis of γH2A.X expression in DLD-1 and HCT116 cells treated with oxaliplatin (40 µM) or a combination with the antioxidant NAC (5 mM) for 24 h. β-Actin was used as a loading control.
