# Supplementary figures and images for: Disrupting G6PD-mediated Redox homeostasis enhances chemosensitivity in colorectal cancer
Source: Oncogene. 2017 Jul 10;36(45):6282–92. doi: 10.1038/onc.2017.227 (PMC5684443; doi:10.1038/onc.2017.227)

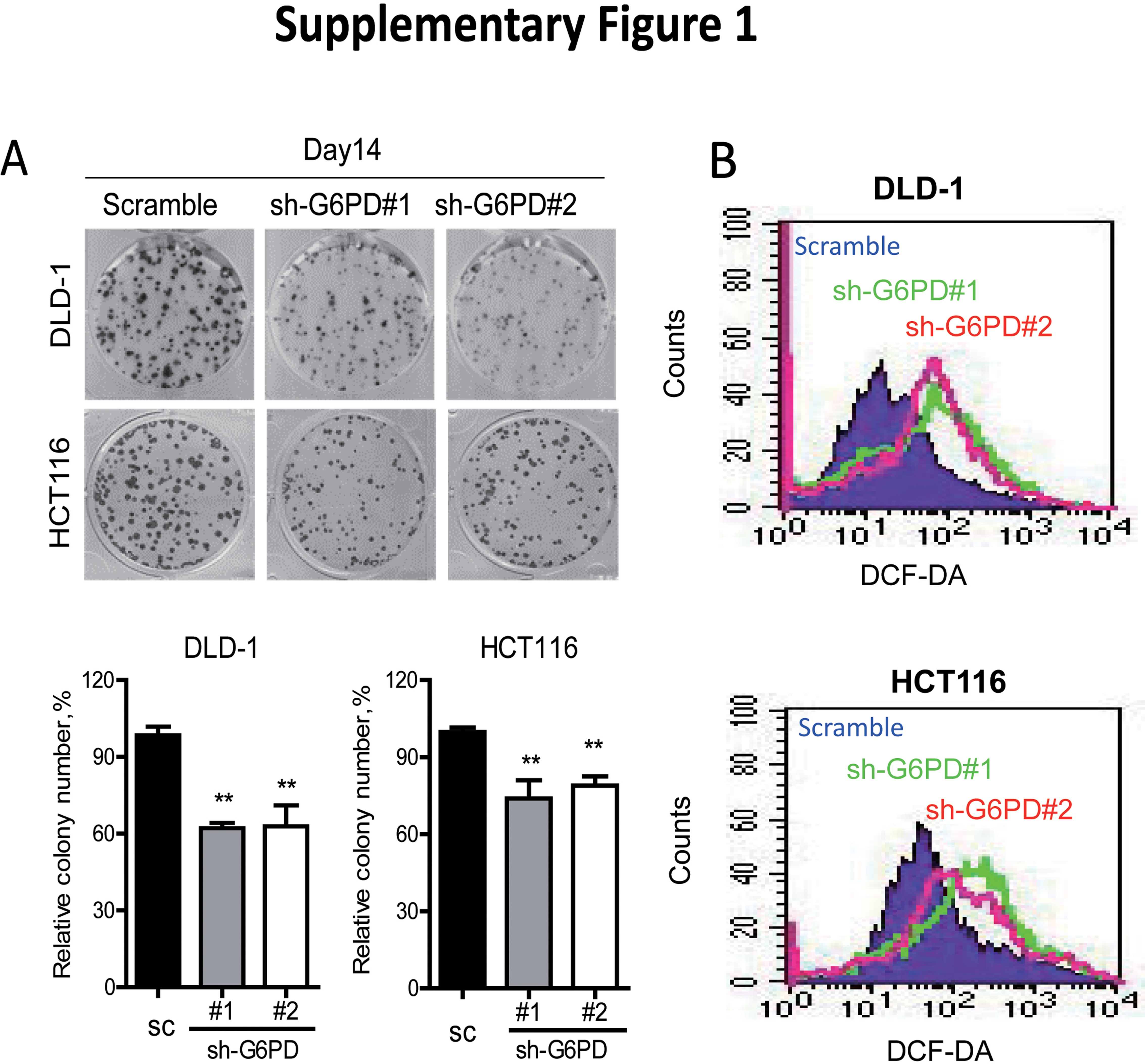

Supplement: Supplementary Figure 1 [file onc2017227x2.tif]

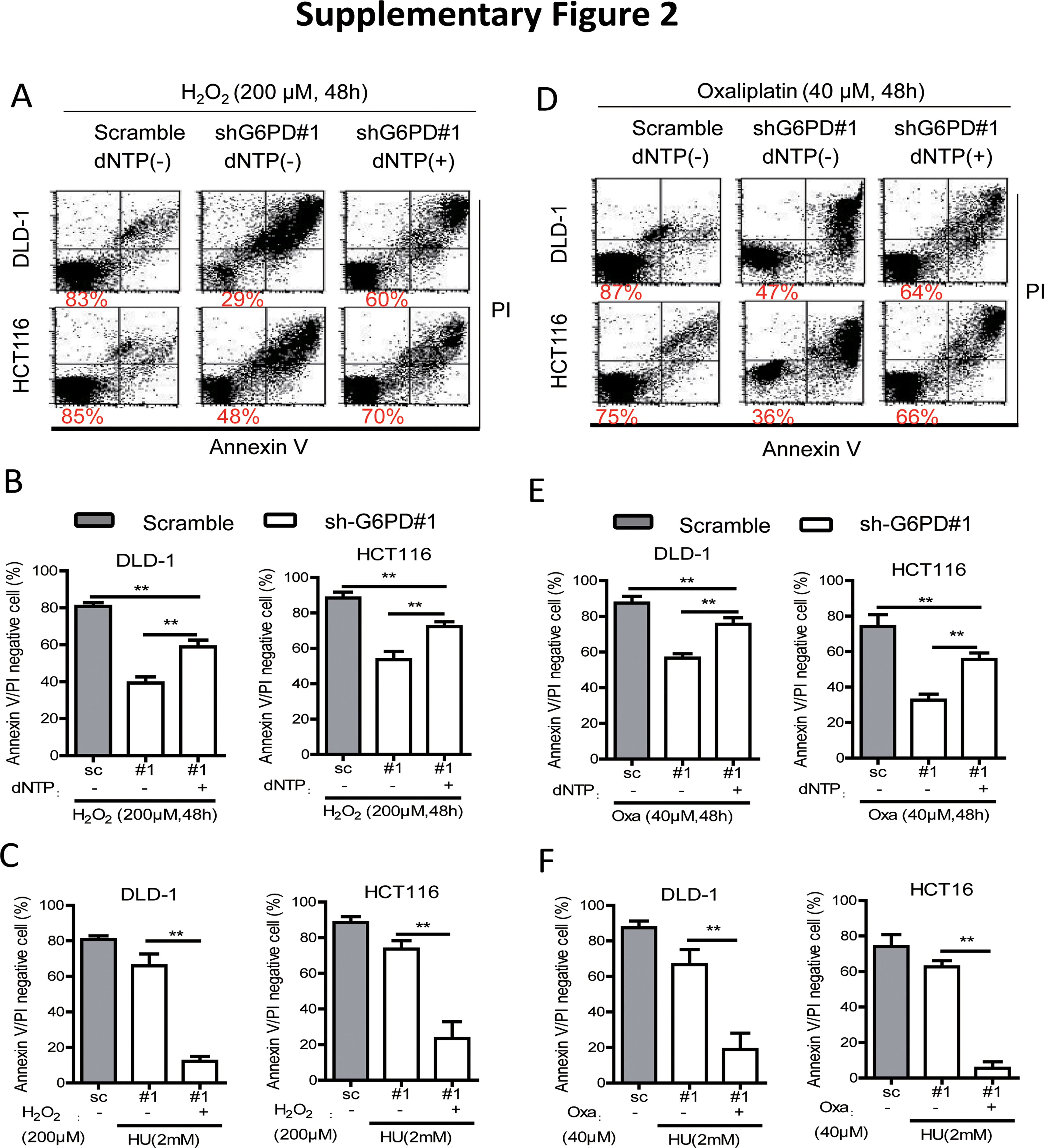

Supplement: Supplementary Figure 2 [file onc2017227x3.tif]

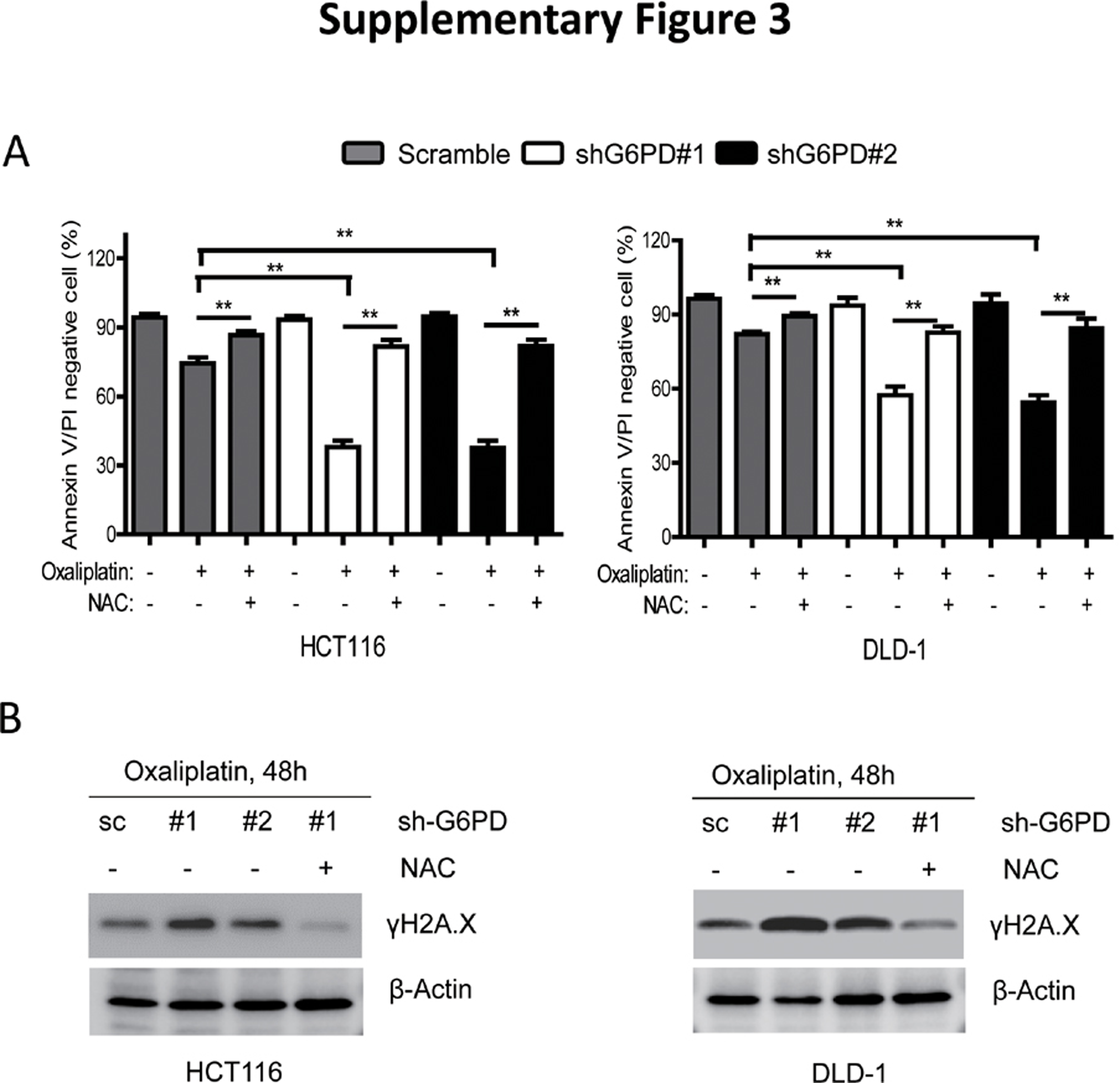

Supplement: Supplementary Figure 3 [file onc2017227x4.tif]
